# The evolution of the socio-cultural and religious characteristics of cancer patients in Morocco: case of the National Institute of Oncology Rabat

**DOI:** 10.1186/s12885-021-08175-y

**Published:** 2021-05-07

**Authors:** Fahd Elkhalloufi, Saber Boutayeb, Fouzia Mamouch, Latifa Rakibi, Sanae Elazzouzi, Hassan Errihani

**Affiliations:** 1 Faculty of Medicine and Pharmacy, Mohammed V University, Rabat, Morocco; 2 Department of Medical Oncology, National Institute of Oncology, Rabat, Morocco

**Keywords:** Cancer, Socio-cultural characteristics, Religious characteristics

## Abstract

**Background:**

In 2020, Morocco recorded more than 59,370 new cases of cancer and more than 35,265 cases of death (International Agency for Research on Cancer, Annual report Morocco, 2020). Cancer is always accompanied by socially constructed, differentiated, and contingent interpretations and practices according to the socio-cultural and religious characteristics of each region. The study aims at describing the evolution of the socio-cultural and religious aspects of Moroccan cancer patients followed at the National Institute of Oncology (NIO) of Rabat between 2010 and 2020.

**Methods:**

We have prospectively studied all cancer cases diagnosed at the National Oncology Institute (NIO), Rabat in 2019. We have collected 1102 cases. The data collected was compared with the results of the study carried out in 2010 (1600 cases). Statistical analysis has been assessed by SPSS 20 software and the correlations between socio-cultural characteristics were examined using a chi-square test.

**Results:**

From a socio-economic point of view, almost all patients claim that cancer is a costly disease as well as a disease that leads to a drop in income and the inevitable impoverishment of Moroccan patients. The illiteracy rate is still high; rising from 38% in 2010 to 42.80% in 2020. On the psychological level, damage to body image (alopecia, mastectomy, hysterectomy,) can lead to stigmatizing and harms the marital relationship. The number of patients experiencing divorce and marital separation that seems to occur following cancer pathology remains high, despite a decrease of nearly 50% between 2010 and 2020. Concerning the spiritual aspect, in the Arab-Amazigh-Muslim culture, the impact of the occurrence of cancer is very particular, and the repercussions are assessed differently depending on the degree of conviction. For practicing believers, cancer is considered a divine test and an opportunity to improve. In the Qur’an, God tests the best of his disciples to reward them The rate of practicing believers has evolved from 49% in 2010 to 85.50% in 2020.But for non-practicing believers, cancer is regarded as a divine punishment coming from outside. New behaviors reported by this research concern the use of “roquia”. This spiritual cure is considered as an anti-cancer remedy. It uses Allah’s words from the holy “qur’an”, his faires names and his attributes. 42% of patients use “roquia”. Concerning phytotherapy, there was an increase in the percentage of participants using medicinal plants and even the most harmful plants (Arestiloch, Euphorbia) from 26% in 2010 to 51.50% in 2020.

**Conclusion:**

The precarious social level of cancer patients, the lack of social and medical coverage, illiteracy, and lack of knowledge of religion, as well as dissatisfaction with conventional medicine, may lead patients to the use of traditional medicine (medicinal plants, visit of “marabouts”, “roquia”). This can have a negative impact on the quality of access to oncology care.

## Background

In Morocco, cancer is the second leading cause of death, with an estimated incidence rate of 148.30 cases per 100,000 and an estimated mortality rate of 86.90 cases per 100,000. In 2020, Morocco recorded more than 59,370 new cases of cancer. Breast cancer (19.80%) and lung cancer (12.40%) were the most common. On the other hand, 35,265 cases of death were recorded [1].

Cancer is accompanied by socially constructed interpretations and practices, differentiated according to the socio-cultural characteristics of each region [2]. Indeed, in Moroccan culture, cancer is synonymous with death [3]. Patients face many challenges [4]. These include family difficulties related to uncertainty about the place and social role or separation from the spouse, and social and professional difficulties related to access to care, social and medical coverage [5].

Several studies conducted at the National Institute of Oncology (INO) in Rabat [2, 6, 7] have shown that certain socio-cultural characteristics can have harmful consequences on the patient and his family environment. 64% of patients have low incomes, 87% are unemployed and 83% have no medical coverage covering the cost of care. This requires doctors to adapt treatment protocols to accord with the financial means and stocks available in the hospital, and not to therapeutic standards that include increasingly expensive drugs (targeted therapies and new chemotherapy drugs).

This has a psychological impact due to the fear of ineffective treatment (fear, anxiety). Difficulties in communication between caregivers and patients, due mainly to illiteracy and the Amazigh dialect, generate not only create anxiety for the patient, but also for the physician, who must ensure therapeutic care alone without the patient being involved. On the other hand, women have a high rate of divorce as a result of cancer. Besides, a survey of sexuality and cancer had revealed that 85% of couples had sex-related disorders.

Other practices further exacerbate this finding and jeopardize the process of treating patients. These include the visit of “marabouts” and the use of harmful plants that endanger the patient’s life, or extremism in the practice of religion in some cancer patients.

With this in mind, this study aims at describing the evolution of socio-cultural and religious characteristics of cancer patients between 2010 and 2020.

## Methods

This is a comparative cross-sectional study that aims to describe the evolution of the socio-cultural and religious aspects of Moroccan cancer patients followed at the NIO of Rabat between 2010 and 2020. NIO is the leading oncology center in Morocco (in operation since 1985) recruiting more than six thousand new patients per year. Meanwhile, the hospital is a center of reference in oncology in Morocco. The study took place between January 2019 and January 2020.

### Inclusion criteria

Patient recruitment concerns all patients followed at NIO who have given their written consent to the study through a consent form; must be at least 18 years of age, histologically confirmed cancer regardless of its stage and current or completed treatment.

Before patient recruitment, the descriptive study was approved by the ethics committees of the Faculty of Medicine and Pharmacy of the flap under No. 26/19.

### Exclusion criteria

Patients who do not meet the inclusion criteria above-mentioned are excluded from this research, as well as those with diffuse brain metastases that may have an impact on language or intellectual abilities that bias the questioning.

### Data collection

For data collection, we adopted a questionnaire with questions adapted to the main psycho-socio-cultural characteristics of Morocco. The questionnaire was developed in consultation with a multidisciplinary medical team consisting of a psychologist, a sociologist nursing executives, and medical oncologists.

### Data analysis

The data were analyzed using SPSS software and the bibliographic management using ZOTERO software. Qualitative data were summarized by absolute numbers and percentages. Quantitative data were summarized by medians. The statistical analysis consisted of descriptive analysis of the 1102 data and a study of the relationships between the different characteristics.

## Results

One thousand one hundred and twenty-five (1125) cancer patients were enrolled in this study. Of these patients, 23 were ineligible. The reasons for patient ineligibility were as follows: five [8] subjects were in palliative care, 4 subjects did not have sufficient knowledge of the Arabic language, 8 subjects found the questionnaire long enough to answer all the items, and 6 patients did not have time because of the distances they traveled (exhaustion, fatigue).

As a result, one thousand one hundred and two (1102) patients were included in this study. For patients from 2010, 1600 patients were included in this study.

According to the results obtained, in 2020 women represent 78% of the sample, while in 2010 women represent 57.20%. Regarding marital status, married patients predominate with 73.80% in 2020 and 52% in 2010. The divorce rate was almost declined by 50% (17% in 2010 and 8.30% in 2020). The age of the participants varies between 20 and 92 years. The median age is 51. The most affected age group is 40–60 (59.62%), of which 85% are women.

Islam is the official religion (either in 2010 or 2020). Compared to ethnic groups, the rate of participants of Amazigh origin has decreased by 18.10% in 2020 and 25% in 2010 in addition to an increase for nationals of Arab origin (75% in 2010 and 81.90% in 2020).

There is also an increase in the use of the Arabic language (75% in 2010 and 92.40% in 2020) as a spoken language: The spoken language is the Moroccan dialect called “darija”, that is to say [9]. It is generally used as a communication tool between Arabic and Amazigh speakers.

The illiteracy rate remains high (38% in 2010 and 42.80% in 2020). There is also an improvement for participants arriving at secondary school or higher (7% in 2010 and 18.40% in 2020). The educational situation at the rural level is much more deleterious.

### Socio-economic characteristics

According to Table 1, 58.20% of patients have no income, 28.70% receive less than the SMIG (legal minimum wage in Morocco), which is $270 per month [10], and only 13% of patients receive a salary above the SMIG. Nevertheless, in 2010, 76% of patients received less than the SMIG.

**Table 1:** Socio-economic characteristics of cancer patients in Morocco

Year	2010 (1600 cases)	2020 (1102 cases)
Variable		
	Number of cases %	Number of cases %
**Sex**		
Man (male)	685 (42,80%)	242 (22,00%)
Woman (female)	915 (57,20%)	860 (78,00%)
**Marital Status**		
Single	400 (25,00%)	122 (11,10%)
Married	832 (52,00%)	813 (73,80%)
Divorced	272 (17,00%)	093 (08,30%)
Widowed	096 (06,00%)	075 (06,80%)
**Ethnic Group**		
Arab	1200 (75,00%)	902 (81,9%)
Amazigh	400 (25,00%)	200 (18,1%)
**Spoken Langauge**		
Arabic	1120 (75,00%)	1018 (92,4%)
Amazigh	480 (25,00%)	0082 (7,40%)
Frensh or English	000 (00,00%)	0002 (0,20%)
**Level of education**		
Illiterate	608 (38,00%)	472 (42,80%)
Qurranic	560 (35,00%)	195 (17,70%)
Primary	320 (20,00%)	232 (21,10%)
Secondary	048 (03,00%)	155 (14,10%)
University	064 (04,00%)	048 (04,30%)
**Social Security**		
No insurance	1328 (83,00%)	724 (66,20%)
With Inssurance	192 (12,00%)	354 (32,10%)
Paid	080 (05,00%)	019 (01,70%)
**Patient’s income**		
No income	0224 (14,00%)	643 (58,30%)
Les than 270 $	1216 (76,00%)	317 (28,70%)
270 $ and more	0160 (10,00%)	142 (13,00%)
**Provenance**		
region of Rabat	0320 (20,00%)	347 (31,50%)
other region	1280 (80,00%)	755 (68,50%)
**Place of residence**		
Urbain	0400 (25,00%)	784 (71,10%)
Rural	1200 (75,00%)	318 (28,90%)
**Family support**		
Yes	Not known	857 (77,80%)
`Non	Not known	245 (22,20%)

For basic medical coverage, there is a decline in the rate of patients with no insurance from 83% in 2010 to 66.20% in 2020. In this case, the State shall bear the costs of treatment within the limits of the treatments available in public health establishments. At the same time, there has been an increase in the percentage of participants affiliated with Health Insurance from 12 to 32.10% between 2010 and 2020. This mode of insurance is based on the principles of social insurance for the benefit of persons engaged in a remunerated activity (government employees and private sector employees).

Concerning the geographical remoteness of patients. In 2020, 68.50% of patients come from outside the city of Rabat and 28, 90% from rural areas. In 2010 80% of patients come from outside the city of Rabat, 70% of them from rural areas. To access care, NIO patients travel a distance with a median of 80.00 km in 2020 and 200 km in 2010. This remoteness imposes expenses for transportation as well as hotel accommodation during treatment or while waiting for an appointment. Nearly 70% of patients report significant transportation costs exceeding an average of 30 $ per treatment session. Add to this the cost of hotels and food. As a result, 66% of patients turn to their families or associations to ensure their care and/or stay.

### Marital and family relationship

On the marital level, the divorce rate decreased from 17% in 2010 to 7.30% in 2020. It should be noted that 97% of divorced participants are women. At the family level, Moroccan society is a solidarity society; solidarity and family support are present in 77, 80% of Moroccan patients. 45% of them received material assistance. Family support for the patient is essential for his or her adaptation to the disease and treatment, disease management and quality of life [11].

According to the results obtained. In Table 2, the rate of practicing believers rose from 49% in 2010 to 85.50% in 2020. The impact of cancer onset varies according to the degree of practice [12]. Among religious believers (85.50%), cancer is a divine test. It has therefore led to an acceptance of the illness or even pride in being chosen by God. More than half of them become more practicing as a result of their cancer. This behavior is manifested in prayer, reading the Qur’an, and praising God. Some patients adopt rather harsh religious behaviors, even against medical advice, such as fasting (3.40%), going on a large or small pilgrimage (0.40%).

**Table 2:** Religious characteristics related to cancer

Year	2010 (1600 cases) Number of cases (%)	2020 (1102 cases) Number of cases (%)
Variable		
**Religious customs**		
Non practicing believer	816 (51,00%)	160 (14,50%)
Practicing believer	784 (49,00%)	942 (85,50%)
**Wearing the Veil**		
Yes	1088 (68,00%)	755 (68,50%)
No	0512 (32,00%)	347 (31,50%)
**Visit the marabouts**		
No	1296 (81,00%)	1029 (93,40%)
Yes	0304 (19,00%)	0073 (06,60%)
**Medical Plants**		
No	1184 (74,00%)	534 (48,50%)
Yes	0416 (26,00%)	568 (51,50%)
**Alcohol and Tobacco cessation**		
No	0040 (23,50%)	064 (22,00%)
Yes	0128 (76,50%)	227 (78,00%)
Healthy person	1432 (89,50%)	811 (73,60%)
**Roquia**		
No	Not known	643 (58,30%)
Yes	Not known	459 (41,70%)

Nevertheless, among the non-practicing, cancer is due to divine punishment. Therefore a sense of guilt among the study population is ingrained in their psyche, as deserve the label of “bad Muslims”. This way of thinking engenders an extremist religious practice, and in some cases, this practice goes against medical recommendations (fasting, pilgrimage during treatment). In the two groups studied, more than 68% of participants woman systematically wear the veil, either to cover alopecia related to different therapies or to get closer to God.

In both groups, some patients stopped tobacco and drinking alcohol. In 2010, 76.50% of the patients stopped this behavior after the discovery of the disease. All of them were men. The same pattern is observed in 2020; 78% of the patients declared quitting these habits.

In addition, “roquia” now appears as an emerging new phenomenon espoused by cancer patients. Nearly 42% of patients use “roquia” as a remedy. This Spiritual therapy as an unconventional therapeutic practice appears and spreads in a context favored by the religious effervescence associated with the return to an “original” Islam [13]. It is a method of cure based on reading the quranic verses and recitations of the prophet “Sunnah” [14]. The “roquia” is what is said for the purpose of seeking protection and treating cases of illnesses, such as fever and epilepsy. The “roquia” uses Allah’s words from the holy “Qur’an”, his faires names and his attributes. Also the “roquia” uses the above mentionned in addition the dihkr (the words in allah’s remembrance) and established prayers [15].

Similarly, another aspect of Moroccan culture is added to “roquia”. This is the visit of the “marabouts”. It would have a divine power “albaraka” for those who practice it, whether for the treatment of incurable diseases, mental illnesses, or protection from the evil eye [14]. In our context, 6.60% of participants do visits to the “marabouts” compared to 19% in 2010.

Regarding medical Plants, there has been an increase in the percentage of participants using medicinal plants, from 26% in 2010 to 51.50% in 2020. Dozens of plants are thus used in different forms (plant extract, fumigation, mixing with other plants,). The most used plants according to our survey are Saffron, Propolis, Thyme, Oregano, Myrtle, Cypress, Arar, Eucalyptus, Dill, Sagebrush/ Armoise/ Mugwort, *Potentilla anserina*, Lavender. There are even the most harmful plants (Arestiloch, Euphorbus). More than half of those interviewed use plants mentioned in the Qur’an and the Sunnah: honey, dates, pomegranate and its leaves, nigella, garlic, ginger, olives, and figs.

## Discussion

Cancer leads to a disruption in the patient’s life [16]. Different spheres of life are affected: social, family, marital and professional life [17]. Indeed, the evolution of the socio-economic level of patients, from 2010 to 2020 was always negative. Almost all patients claim that cancer is a costly disease, which in addition, leads to a drop in income and the inevitable impoverishment of Moroccan patients [2]. It has also been noted that the illiteracy rate is still high, rising from 38% in 2010 to 42.80% in 2020. This rate is four times higher among women than among men, which leads to a greater lack of knowledge of medical information by women.

Culturally, cancer is associated with death [4]. It raises questions concerning finitude and death very soon [18]. It causes a “bad death” for all those it affects [19]. This can lead to the stigmatization of people with cancer [20]. This phenomenon appears in several forms. First, damage to body image (alopecia, mastectomy, hysterectomy) can lead to stigmatizing behavior from the immediate environment [17] and avoidance of all social contact and leisure activities [21]. Second, the psychological impact of breast cancer has a dual origin: on the one hand, it is linked to the image of cancer, which signifies suffering, death, and on the other hand, to the image of breasts as a symbol of femininity, motherhood, and sexuality [17].

This harms the marital relationship, as Moroccan women are often dependent on their husbands to cover the costs of care [2]. This social, legal, and economic vulnerability is the cause of greater suffering and distress for a Moroccan cancer patient compared to a male patient. This is illustrated by the large number of female patients who are victims of divorce and separation despite a decrease of almost half of cases between 2010 and 2020. In contrast, in the case of men with cancer, they are often supported by their wives.

On the spiritual level, Cancer is an opportunity to challenge lifestyle and values [22]. It is not surprising that spirituality positively influences health or well-being if it is defined in positive terms [23]. In the Arab-Berber/ Amazigh-Muslim culture, the impact of the onset of cancer is assessed differently according to the degree of belief [2].

For practicing believers, cancer is considered a divine test. It is attributed to the will of the Almighty (God, genius, spirit, etc.) who calls to order those who have strayed from the “right path” by sending them this trial [24]. In the “Qur’an”, God tests the best of his disciples to reward them, and the result is an acceptance of illness and even pride in having been chosen by God [2].

In our study, we see an increase in the rate of practicing believers, from 49% in 2010 to 85.50% in 2020. Therefore, feeling supported by one’s faith may alleviate the symptoms of depression and anxiety [24]. López and his team [25] showed the place of spirituality in the acceptance of gynecological cancers in women with gynecological cancers that had a high level of spiritual well-being. Paola Lissoni [26] demonstrated a relationship between the degree of spiritual faith and the response to chemotherapy in patients with advanced cancer. Tumour regression, lymphocyte count, and three-year survival were all significantly higher in patients with a high faith score.

However, another audience views the disease as divine punishment from the outside [20]. It is not uncommon to find that patients attribute their disease to an “educational God” [27]. Divine punishment seems to be particularly prevalent among cancer patients [28].

This new spiritual therapy, “roquia”, adopted by some patients, is based on recitations of Qur’anic verses and the invocations taught by the Prophet Muhammad [14]. The patient can treat himself or herself with the help of a therapist of roquia (Raqi). Its users believe that it is effective in dealing with incurable diseases of which cancer is one example**.** For the Raqis when certain symptoms persist after the patients visited a therapist, who uses modern medicine, they infer that the evil eye or the devils are responsible for that disease [13].

This mode of treatment is not immune to adverse complications in the use of medicinal plants with “roquia” [13]. Indeed, many “Raqis” sometimes draw from the traditional knowledge of plants called remedy of the Arabs “dwa al-‘arab”, ([29]: 65). For example, they may use harmel (*Peganum harmala*) which is a powerful purgative toxic to the body [30].

The use of saltwater or seawater with the recitation of the Qur’an is considered a practice that has a purifying virtue without forgetting the danger of salt for hypertensive people. Some “Raqis” use senna leaves ([31] or jujube leaves (sedra, *Ziziphus lotus*) as strong purgatives while they are toxic for the body [32]. But the most serious is when some patients abandon their treatments, convinced that their ailments can be treated with Roqya [13].

In addition to the plants mentioned, others are used as complementary or anticancer treatments, which doubled in the last decade between 2010 and 2020. Popular beliefs assume that plants are healthier “natural” and can be effective in chemotherapy [33]. Plants are also responsible for a large number of drugs, with fewer side effects.

However, the simultaneous use of these plants is not immune from harmful effects [34]. Medicinal plants may contain toxic substances such as mercury, lead, copper, iron, manganese, nickel, zinc, and arsenic, which can cause serious complications [35]. This is the case of aristolochia (Aristolochia longa L). This species contains aristolochic acid which can cause irreversible renal damage with hematuria and limb paralysis [36]. In addition “*Euphorbia resinifera*”, whose vernacular name is “Daghmous” is widely used to treat cysts [8]. In [37], several cases of intoxicated patients have been reported by the Moroccan Centre for Pharmacovigilance by ingestion of the “Daghmous”. Wild garlic (*Allium ursinum*) does not contain harmful substances but may be confused with poisonous plants [38]. Other effects have been reported, such as acute encephalopathy (in case of overconsumption of saffron), tubulointerstitial nephritis, liver damage, digestive disorders (diarrhea, vomiting, constipation), rectal bleeding [39].

According to Moroccan culture, the visit of “marabouts” has a divine power ‘albaraka’ where “marabouts” are found for the treatment of incurable diseases or for the treatment of mental illnesses, and others for the treatment of infertility or to protect against the evil eye [14].

This behavior is experiencing a net decrease from 19% in 2010 to 6.60% in 2020. This can be explained by the referral of patients to the “roquia” rather than to the visit of “marabouts”, who ask for a lot of financial means ranging from simple gifts to animal sacrificial offerings “sadaka or lahdya” (rooster, sheep, cloth, money..etc.) [40].

### Strengths and limitations

The strengths of this study lie primarily in the sample size of 1102 participants, so the study population was well characterized and more than 70 characteristics were worked on. The results are not necessarily generalizable since the study site is limited to patients followed at NIO in Rabat. This research concerns the muslim community. it is not generalized to other Christian or Jewish communities. In this study we were limited to the investigation by questionnaire. The comparison between 2010 and 2020 is only for associated socio-cultural and religious characteristics.

## Conclusion

The evolution of the socio-cultural and religious characteristics of Moroccan cancer patients shows a set of difficulties that hinder a better quality of access to oncology care. Indeed, the precarious social level of cancer patients, the lack of social and medical coverage, illiteracy, the upset psychological state (stigmatization, lack of self-esteem), and lack of knowledge of religion, as well as dissatisfaction with conventional medicine, may lead patients to the use of traditional medicine (medicinal plants, visit of “marabouts”, “roquia”) [39].

All these practices are left to the legislative, scientific, and academic oblivion. This can have harmful complications on the health of patients. As a solution, first of all, the generalization of basic medical coverage, as well as the generalization of social coverage, can ensure access to oncology care. Medicinal plants must obey strict standard rules that only the specialist in herbal medicine can meet. This necessarily implies the regulation of the profession in our country Morocco. The use of “roquia” or marabou visits must be controlled by the state. The involvement of the family circle, as a care partner, can also play an important role in the care of the patient.

## Data Availability

The datasets generated and/or analysed during the current study are not publicly available due [The data of participating patients remain confidential and anonymous in the informed consent with the patient. All patients’ data are available and archived in our institute] but are available from the corresponding author on reasonable request.

## Abbreviations

IARC: International agency for research on cancer
NIO: National institute of oncology
SMIG: Salaire minimum légal
WHO: World health organization
SPSS: Statistical Package for Social Science
AMFROM: the Moroccan association for training in medical oncology
